# DEGRO practical guidelines for radiotherapy of breast cancer VI: therapy of locoregional breast cancer recurrences

**DOI:** 10.1007/s00066-015-0939-7

**Published:** 2016-03-01

**Authors:** Wolfgang Harms, W. Budach, J. Dunst, P. Feyer, R. Fietkau, W. Haase, D. Krug, M. D. Piroth, M.-L. Sautter-Bihl, F. Sedlmayer, R. Souchon, F. Wenz, R. Sauer, Breast Cancer Expert Panel of the German Society of Radiation Oncology (DEGRO)

**Affiliations:** 1 Abteilung für Radioonkologie, St. Claraspital, Kleinriehenstrasse 30, 4016 Basel, Switzerland; 2 Heinrich-Heine-University, Duesseldorf, Germany; 3 University Hospital Schleswig-Holstein, Kiel, Germany; 4 Vivantes Hospital Neukoelln, Berlin, Germany; 5 University Hospital Erlangen, Erlangen, Germany; 6 Formerly St.-Vincentius-Hospital, Karlsruhe, Germany; 7 University Hospital Heidelberg, Heidelberg, Germany; 8 HELIOS-Hospital Wuppertal, Witten/Herdecke University, Wuppertal, Germany; 9 Municipal Hospital, Karlsruhe, Germany; 10 Paracelsus Medical University Hospital, Salzburg, Austria; 11 Formerly University Hospital Tuebingen, Tuebingen, Germany; 12 University Medical Center Mannheim, Medical Faculty Mannheim, University of Heidelberg, Mannheim, Germany

**Keywords:** Chest wall recurrence breast cancer, Axillary recurrence breast cancer, Supraclavicular recurrence breast cancer, Mastectomy, Reirradiation, Brustwandrezidiv Mammakarzinom, Axilläres Rezidiv Mammakarzinom, Supraklavikuläres Rezidiv Mammakarzinom, Mastektomie, Rebestrahlung

## Abstract

**Objective:**

To update the practical guidelines for radiotherapy of patients with locoregional breast cancer recurrences based on the current German interdisciplinary S3 guidelines 2012.

**Methods:**

A comprehensive survey of the literature using the search phrases “locoregional breast cancer recurrence”, “chest wall recurrence”, “local recurrence”, “regional recurrence”, and “breast cancer” was performed, using the limits “clinical trials”, “randomized trials”, “meta-analysis”, “systematic review”, and “guidelines”.

**Conclusions:**

Patients with isolated in-breast or regional breast cancer recurrences should be treated with curative intent. Mastectomy is the standard of care for patients with ipsilateral breast tumor recurrence. In a subset of patients, a second breast conservation followed by partial breast irradiation (PBI) is an appropriate alternative to mastectomy. If a second breast conservation is performed, additional irradiation should be mandatory. The largest reirradiation experience base exists for multicatheter brachytherapy; however, prospective clinical trials are needed to clearly define selection criteria, long-term local control, and toxicity.

Following primary mastectomy, patients with resectable locoregional breast cancer recurrences should receive multimodality therapy including systemic therapy, surgery, and radiation +/− hyperthermia. This approach results in high local control rates and long-term survival is achieved in a subset of patients. In radiation-naive patients with unresectable locoregional recurrences, radiation therapy is mandatory. In previously irradiated patients with a high risk of a second local recurrence after surgical resection or in patients with unresectable recurrences, reirradiation should be strongly considered. Indication and dose concepts depend on the time interval to first radiotherapy, presence of late radiation effects, and concurrent or sequential systemic treatment. Combination with hyperthermia can further improve tumor control.

In patients with isolated axillary or supraclavicular recurrence, durable disease control is best achieved with multimodality therapy including surgery and radiotherapy. Radiation therapy significantly improves local control and should be applied whenever feasible.

## Abstract (de)

**Ziel:**

Aktualisierung der Strahlentherapieleitlinien bei Patienten mit lokoregionalen Mammakarzinomrezidiven, basierend auf der aktuellen S3-Leitlinie.

**Methoden:**

Es erfolgte eine umfassende Recherche der wissenschaftlichen Literatur mit den Suchbegriffen: „lokoregionales Mammakarzinomrezidiv“, „Thoraxwandrezidiv“, „Lokalrezidiv“, „regionales Rezidiv“ und „Mammakarzinom“, eingeschränkt durch die Begriffe „klinische Studie“, „randomisierte Studie“, „Metaanalyse“, „systematischer Review“ und „Leitlinie“.

**Schlussfolgerungen:**

Patienten mit isolierten In-Brust- oder regionalen Rezidiven sollten mit kurativer Intention behandelt werden. Bei Patienten mit einem ipsilateralen In-Brust-Rezidiv ist eine Mastektomie die Standardbehandlung. Bei einem Teil der Patienten ist eine erneute Brusterhaltung mit anschließender Teilbrustbestrahlung eine angemessene Alternative zur Mastektomie. Nach erneuter Brusterhaltung sollte eine Bestrahlung obligat durchgeführt werden. Die größten Erfahrungen zur Rebestrahlung existieren für die Multikatheter-Brachytherapie. Dennoch sind prospektive Studien notwendig, um Selektionskriterien sowie Langzeittoxizität und -kontrollraten genauer zu bestimmen.

Patienten mit resektablen lokoregionalen Mammakarzinomrezidiven nach primärer Mastektomie sollten mit einem multimodalen Therapiekonzept mit kompletter Resektion, systemischer Therapie und Bestrahlung +/− Hyperthermie behandelt werden. Hierdurch können hohe lokale Kontrollraten und in einem Teil der Patienten lange Überlebenszeiten erreicht werden. Eine Strahlentherapie sollte obligat bei bisher nicht bestrahlten Patienten mit inoperablen lokoregionalen Rezidiven durchgeführt werden. Bei vorbestrahlten Patienten mit einem hohen Rezidivrisiko nach chirurgischer Resektion oder einem inoperablen Rezidiv sollte eine Rebestrahlung dringend in Erwägung gezogen werden. Indikationsstellung und Dosiskonzepte sind vom Intervall zur ersten Bestrahlung, der Präsenz und dem Ausmaß später Strahlenreaktionen sowie von parallelen/sequenziellen systemischen Therapien abhängig. Eine Kombination mit einer zusätzlichen Hyperthermie kann die Tumorkontrolle weiter verbessern.

Bei Patienten mit isolierten axillären oder supraklavikulären Rezidiven kann eine dauerhafte Krankheitskontrolle am besten mit einem multimodalen Behandlungskonzept bestehend aus Chirurgie und Radiotherapie erreicht werden. Eine Strahlentherapie sollte, wann immer möglich, durchgeführt werden, da sie zu einer signifikanten Verbesserung der lokalen Kontrolle führt.

## Introduction

Treatment of locally recurrent breast cancer remains an interdisciplinary challenge, since treatment options are limited or at least restricted due to previous treatments. Data from large randomized trials have demonstrated that locoregional recurrences occur in approximately 5–15 % of patients, despite them having received adjuvant radiotherapy after primary mastectomy or breast-conserving surgery (BCS; [1–4]). The most common site of recurrence after adjuvant radiotherapy is the ipsilateral breast or chest wall, comprising 60–95 % of all locoregional events [5–7]. Locoregional recurrences are typically associated with an increased risk of concurrent or subsequent systemic relapses [8, 9]. Particularly early recurrences within the first 2 years after primary treatment seem to have a worse prognosis [9]. As previous guidelines from the German Society of Radiation Oncology (DEGRO) expert panel [10–12] focused on primary treatment of breast cancer, the current publication addresses radiotherapeutic options for locoregional recurrences.

## Therapy of ipsilateral breast tumor recurrence

**Statement of the** **German S3 Guidelines 2012** [13]:

**Statement recurrence 1: Local (in-breast) recurrence**

- In patients with invasive or noninvasive in-breast recurrences, optimal local tumor control is achieved with a secondary mastectomy.
- In patients with ductal carcinoma in situ or invasive breast cancer with a long recurrence-free interval and no skin infiltration, a second breast conservation can be considered.
- In the case of a second breast conservation, the possibility of reirradiation (partial breast irradiation, PBI) should be evaluated.
- In the case of a second breast conservation, the patient should be informed about an increased risk of subsequent in-breast recurrence.

BCS followed by adjuvant radiotherapy has become the standard of care for the majority of breast cancer patients. Ipsilateral breast tumor recurrence (IBTR) is diagnosed in approximately 5–10 % of patients at 10 years after breast-conserving therapy (BCT; [10, 14–17]). International guidelines recommend mastectomy as standard treatment for IBTR after BCT [13, 18]. Salvage mastectomy results in locoregional control rates of 69–98 % and 5-year survival rates of 53–85 % [19, 20]. Nevertheless, this approach is not founded on solid data demonstrating a clear advantage of radical surgery over a second attempt at BCT for all IBTR patients [21]. There are no published or ongoing prospective studies comparing mastectomy to repeat BCS in patients with IBTR. Published data are limited and most series reporting on a second conservative approach, with or without the addition of adjuvant radiotherapy, are single-institution retrospective studies. The Radiation Therapy Oncology Group (RTOG) introduced a phase II prospective trial of repeat BCS adding 3D conformal partial breast reirradiation for local recurrence in 2010 [22]. Additionally, mutilating rescue surgery causes patients enormous emotional and physical distress [23]. Furthermore, the currently implemented close follow-up routine has led to detection of IBTR at early stages technically amenable to a second breast conserving approach. Thus, a subgroup of patients remains to be defined who can be safely treated with a second breast conserving approach without oncologic compromises. Unfortunately, no prospective data have been published so far.

### Definition of selection criteria for a second BCS

Veronesi et al. suggested that patients with IBTR constitute a heterogeneous group, with true tumor recurrences and new primary tumors [24]. In a large retrospective study on 1410 patients, Gujral et al. [25] reported a cumulative incidence rate of new primaries after whole-breast irradiation of 0.8, 2, and 3.5 % at 5, 10, and 15 years respectively. The authors concluded that whole-breast irradiation approximately halves the rate of new primaries. Smith et al. [26] reported a significantly improved 10-year overall survival (OS) in patients with new primaries (75 %) compared to patients with true recurrences (55 %) in a retrospective study on 136 patients with IBTR as the first site of failure. This question has been addressed in several studies attempting to differentiate between true recurrences and new primaries [26–29], from which the following conclusions can be derived: the majority of IBTR are true recurrences, which tend to occur earlier and in the same quadrant as the initial tumor, metastasize earlier and more often, and have a shorter overall and disease-free survival (DFS) than new primaries. Further retrospective studies [30–32] evaluating IBTR showed that there are subgroups of patients with improved rates of OS, DFS, and second local recurrence. Based on these data, the DEGRO expert panel suggests possible selection criteria for patients who may be candidates for a second breast-conserving approach (Table 1). However, repeat BCS alone is associated with increased local failure rates ranging between 19 and 38 % (Table 2; [33]). Recent studies showed promising OS rates in selected patients treated with second conservative surgery followed by partial-breast irradiation (PBI; [34, 35]). The following survey provides an overview of different radiation techniques applied for reirradiation after BCS for IBTR (Table 3).

#### The DEGRO expert panel suggests the following selection criteria for a second breast conserving approach

**Table 1:** Possible selection criteria for patients with ipsilateral breast tumor recurrence who may be candidates for a second breast-conserving approach

Isolated ipsilateral breast tumor recurrence
Limited size (< 2–3 cm)
Unifocal disease on ultrasound, mammography, and MRI
Age ≥ 50 years
Long interval between primary treatment and recurrence (≥ 48 months)
Patient preference for a second breast conservation followed by radiotherapy
A second breast conservation is technically feasible and will result in acceptable cosmetic results

### Brachytherapy after BCS for IBTR

The most solid evidence for reirradiation of IBTR exists for brachytherapy (BT). The Groupe Européen de Curiethérapie and the European Society for Radiotherapy & Oncology (GEC-ESTRO) working group reported on a retrospective collaborative analysis of 217 IBRT patients treated between 2000 and 2009 with multicatheter BT in eight European institutions [35]. The median total doses delivered through low dose rate (LDR) and pulsed dose rate (PDR) BT were 46 Gy (range 30–55 Gy) and 50.4 Gy (range 49–50 Gy), respectively, and 32 Gy (range 22–36 Gy; equivalent dose in 2-Gy fractions: 43 Gy_4_) in 5–10 fractions (median 8 fractions) fractions (twice daily) for high dose rate (HDR) BT. With a median follow-up of 3.9 years (1.1–10.3 years) after IBTR retreatment, the 5- and 10-year actuarial second local recurrence rates were 5.6 % (1.5–9.5 %) and 7.2 % (2.1–12.1 %), respectively. The grade 3 and 4 complication rates were 10 % and 1 % (ulceration), respectively. Excellent or good cosmetic results were achieved in 85 %. Further endpoints were the evaluation of survival rates without second IBTR, as well as metastatic recurrence, DFS, and OS. In comparison to salvage mastectomy series, results were reported to be at least equivalent, with 5- and 10-year actuarial rates for metastatic recurrence of 9.6 % and 19.1 %, DFS of 84.6 % and 77.2 %, and OS of 88.7 % and 76.4 %, respectively. Further single-institution studies with small patient numbers support these data [36–39].

### External beam radiotherapy after BCS for IBTR

There is a single report on external beam radiotherapy (EBRT) for treatment of IBTR [40] comprising 39 patients. Retreatment was performed using electrons in single fractional doses of 2 Gy up to 50 Gy to the involved quadrant. At a median follow-up of 51.5 months, 30 women (76.9 %) had an intact breast free of tumor. The RTOG initiated a phase II study of repeat breast-preserving surgery and 3D conformal partial breast reirradiation (PBI) for local recurrence of breast carcinoma with single doses of 1.5 Gy in 15 fractions, twice daily, to a total dose of 45 Gy [22]. The study has reached the accrual goal of 61 patients and is closed, but not yet published.

### Intraoperative radiotherapy after BCS for IBTR

There is only one publication considering intraoperative radiotherapy (IORT) for reirradiation of IBRT [41]. A total of 15 patients were treated by applying IORT with 50-kV X-rays in single dosages of 14.7–20 Gy (applicator surface). At a medium follow-up of 26 months (1–60 months), no local recurrences occurred.

### Toxicity assessment/cosmetic outcome of repeat irradiation for IBTR

In all publications, acute toxicities of the respective method were reported to be low, while the most frequent late reaction pattern was fibrosis. Particularly when assessed by standardized scoring systems, grade 1–2 sequelae in terms of fibrosis, telangiectasia, and/or pain ranged between 44 and 79 % [35, 37, 42]. Severe late reactions, such as skin necrosis or ulceration, were hardly ever stated. In the GEC-ESTRO series of 217 patients [35], 141 patients (65 %) developed late effects: cutaneous and subcutaneous fibrosis (67 %), telangiectasia (16 %), hyperpigmentation (9 %), and ulceration (1 %). Grade 3 and 4 complications were reported in 10  and 1 %, respectively. The cosmetic results were assessed in 109 patients (50.2 %) and rated excellent in 52 (48 %), good in 40 (37 %), fair in 14 (13 %), and poor in 2 (2 %) patients.

#### Conclusions of the DEGRO expert panel for the therapy of ipsilateral breast cancer recurrences

- Although mastectomy is regarded as the standard of care for patients with IBRT, in a subset of patients, PBI after second BCS is an appropriate alternative to mastectomy (Table 1).
- This approach yields high breast preservation rates and does not seem to compromise oncologic safety.
- If a second breast conservation is performed, additional irradiation should be mandatory, particularly in patients who have not received previous irradiation.
- In the case of reirradiation, the largest experience base to date exists for multicatheter BT.
- There is only limited information about the effectiveness of EBRT or IORT, which should be preferentially performed in clinical trials.
- Prospective studies are needed to clearly define selection criteria, long-term local control, and toxicity.

**Table 2:** Outcomes of patients treated with repeat breast conserving surgery alone following ipsilateral breast tumor recurrence

Study/year	Number of Patients	Median follow-up (months)	Local recurrence rate (%)	5-year overall survival (%)
Kurtz [43] 1989	55	51	32	NR
Abner [44] 1993	16	39	31	81
Salvadori [45] 1999	57	73	19	85
Voogd [46] 1999	16	52	38	NR
Ishitobi [47] 2011	78	40	21	NR
Gentilini [48] 2012	161	81	29	84

*NR* not reported

**Table 3:** Outcomes of patients treated with repeat breast conserving surgery and radiotherapy following ipsilateral breast tumor recurrence

Study/year	Number of patients	Median follow-up (months)	RT technique	Repeat RT dose (Gy)	Grade 3/4 toxicity (%)	Local control (%)	Overall survival (%)
Deutsch [40] 2002	39	51.5	EBRT	50	NR	77	78
Kraus [41] 2007	15	26	IORT	20	0	100	93
Chada [38] 2008	15	36	BT	30 or 45	0	89	100
Guix [42] 2010	36	89	BT	30	0	89^a^	97^a^
Kauer [37] 2012	39	57	BT	50	17^b^/0	93	87
Hannoun [35] 2013	217	46.8	BT	LDR 46 PDR 50.4 HDR 32	11	93^a^	76^a^

*NR* not reported, *RT* radiation therapy, *EBRT* external beam radiotherapy, *IORT* intraoperative radiotherapy, *LDR* low dose rate, *PDR* pulsed dose rate, *HDR* high dose rate

^a^10-year actuarial

^b^4 % breast tissue fibrosis and 13 % subjective breast pain

## Treatment of local recurrence after mastectomy

**Statements of the German S3 Guidelines 2012** [13]:

**Statement recurrence 2: Local recurrence after mastectomy**

- An isolated chest wall recurrence should be completely resected (R0).

**Statement recurrence 5: Radiotherapy after surgery for recurrence**

- The indication for radiotherapy after recurrence surgery should be discussed and decided on an interdisciplinary basis. Postoperative irradiation can be applied if no previous irradiation was accomplished or in cases of incomplete resection (R1/R2).
- In inoperable patients, palliative radiotherapy can be reasonable for systemic control.

### Definition and patterns of recurrence

A locoregional breast cancer recurrence after mastectomy is defined as the appearance of tumor in the ipsilateral chest wall, or the axillary, internal mammary, or supraclavicular lymph nodes [8]. The Early Breast Cancer Trialists’ Collaborative Group analysis revealed a 10-year risk of an isolated locoregional recurrence as the first event after mastectomy of 20.3 % for patients with 1–3 positive axillary lymph nodes, and of 32.1 % for patients with ≥ 4 positive nodes. The addition of radiotherapy significantly reduced the risk to 3.8 and 13 %, respectively [2, 49, 50]. In cohorts treated with modern systemic therapies, the total locoregional recurrence rate is substantially lower; however, the relative risk reduction after radiotherapy remains unchanged [51]. Katz et al. [52] reported on local recurrence patterns in a retrospective study of 1031 women treated in five prospective trials with mastectomy and adjuvant chemotherapy without radiation therapy. After a median follow-up of 116 months, the most common sites of isolated locoregional recurrences were the chest wall and the supraclavicular lymph nodes. There is evidence that a local recurrence per se is the strongest predictor for a further recurrence [9].

### Treatment of resectable recurrences in radiation-naive patients

If no previous irradiation has been performed, optimal treatment consists of complete excision of gross disease followed by irradiation [8, 53]. This approach improved local control [54, 55] and may have an effect on survival [53, 56]. Skinner et al. [57] evaluated the effects of dose escalation on local control and survival in 159 patients with an isolated locoregional recurrence after mastectomy. Patients in the standard treatment group were treated to a dose of 50 Gy plus a boost of 10 Gy, while the dose escalation group was treated to a dose of 54 Gy plus a 12-Gy boost. The authors observed a 77 % locoregional control rate (LCR) and a 55 % OS rate at 5 years for the entire group. OS and LCR were not significantly improved in the dose escalation group. In summary, patients with an isolated locoregional recurrence after mastectomy should undergo surgical resection. Postoperative radiation therapy to the chest wall is mandatory and regional nodal irradiation is strongly advised [18, 58]. A standard dose of 50–50.4 Gy with 1.8-/2-Gy fractions should be applied. An additional boost dose of 10 Gy can be applied, particularly if risk factors are present. A further approach to enhance radiation effectiveness is the additional use of hyperthermia. This combination improved clinical response and local control in several phase II studies and randomized trials [59, 60].

For systemic management, endocrine therapy should be administered to all hormone receptor-positive patients in addition to local excision and radiotherapy [61]. In a prospective randomized study, Aebi and colleagues [62] investigated the effects of chemotherapy in 162 patients with completely resected isolated locoregional breast cancer recurrences. These authors found that adjuvant chemotherapy in addition to radiation and endocrine therapy prolonged DFS and OS, particularly in patients with estrogen receptor-negative locoregional relapse. The 5-year DFS and OS rates were 69 and 88 %, respectively. This result challenges the current practice of reluctant use of chemotherapy and provides evidence in favor of offering adjuvant chemo- and radiotherapy to women with completely resected isolated locoregional relapse of breast cancer.

### Treatment of unresectable recurrences in radiation-naive patients

In patients with unresectable isolated locoregional recurrences who have not previously been irradiated, radiation therapy is mandatory. In a retrospective study, Skinner et al. [57] reported that patients with gross disease at the time of radiation had significantly lower 5-year local control (63 %) and survival rates (34 %) compared to patients with no residual disease after surgery or systemic therapy (81 and 62 %, respectively). If complete remission after radiation therapy was accomplished, the 5-year survival rate increased from 27 to 62 % [57]. Therefore, the same recommendations for radiation and endocrine therapy apply as for resectable disease. The boost dose can be increased depending on the size and location of the recurrence. The indication for additional chemotherapy should be defined on an individual basis, since no prospective data are available [63–65]. In summary, multimodality therapy including systemic and radiation therapy has the potential to cure selected patients [9, 53, 66–68].

### Therapy of locoregional recurrences after mastectomy in previously irradiated patients

Treatment options are limited in patients with locoregional recurrences after mastectomy and adjuvant radiotherapy. The optimal sequence of multimodal treatments in this situation has not been evaluated in prospective trials. In current practice, most of these patients are initially treated with systemic therapy, in analogy to neoadjuvant systemic treatment of primarily unresectable breast cancer. If complete resection is possible, surgery should be accomplished; however, “heroic surgery” resulting in large tissue defects or prolonged wound healing problems should be avoided. Traditionally, reirradiation has been used with caution, in fear of an increased normal tissue complication rate. However, a number of small to intermediate size clinical trials have revealed that the grade IV late toxicity after reirradiation with EBRT was within an acceptable range, not exceeding 12 % [5, 69–74]. Simultaneous radiochemotherapy as a treatment option has been investigated in a limited number of trials [63, 64].

### Reirradiation with or without regional hyperthermia

Laramore et al. [69] reported on 13 patients treated with conventionally fractionated electrons for chest wall recurrences. All patients had received previous postoperative chest wall irradiation with doses between 40 and 50 Gy. Of these patients, 62 % were alive and free of local disease after a median follow-up of 12 months. Skin reactions were limited to temporary erythema and dry or moist desquamation. Harms et al. [75] reported on a retrospective study in 58 patients treated with PDR BT molds. The local control rate was 79 %. Grade III fibrosis was experienced by 10 % of the patients and grade IV late effects were suffered by 7 %.

An increasing number of studies on the combination of hyperthermia and reirradiation of the chest wall have been published (Table 4; [59, 60, 71–74, 76, 77]). Jones and colleagues [60] enrolled 109 patients with superficial tumors (70 patients with breast cancer) in a prospective randomized trial comparing irradiation of chest wall recurrences with irradiation and additional hyperthermia. The complete response rate was 66.1 % in the hyperthermia and 42.3 % in the irradiation-only arm. Previously irradiated patients had the greatest incremental gain in complete response: 23.5 % in the non-hyperthermia versus 68.2 % in the hyperthermia arm. No OS benefit was seen. The authors concluded that adjuvant hyperthermia resulted in a significant local control benefit in patients with superficial tumors receiving radiation therapy. These data are supported by a meta-analysis of five randomized trials including 306 patients with advanced primary or recurrent breast cancer [59]. The complete remission rate was significantly improved in patients treated with combined radiation and hyperthermia compared to radiation alone (59 vs. 41 %). OS was not improved.

More recent data were published in a retrospective analysis of 198 patients who underwent either R0 (*n* = 107) or R1 resection (*n* = 91) for recurrent breast cancer. Hyperthermia was used as an adjunct to reirradiation (eight 4-Gy fractions; [73]). After a median follow-up of 42 months, the 5-year locoregional control rate was 78 %. The 5-year grade III/IV late toxicity rate amounted to 11.9 % (*n* = 15 skin ulcerations, *n* = 5 osteoradionecrosis of the ribs). The same working group investigated 248 patients with a macroscopic breast cancer recurrence treated with reirradiation and hyperthermia [78]. After a median follow-up period of 32 months, 70 % of patients had a complete remission. The 5-year local control rate was 39 %. Thermal burn was developed by 23 % of patients, which healed with conservative measures. The incidence of 5-year late grade 3 toxicity was 1 %.

**Table 4:** Outcomes of patients with locoregional chest wall recurrences treated with repeat irradiation +/− hyperthermia

Study	Patients n=	Initial dose (Gy)	RT technique	Repeat dose (Gy)	Complete remission (%)	Toxicity grade (%)
Delanian [79]	11	45–65	BT	60	81.8	II/III 45 IV 9
Harms [75]	58	36–70	BT	2 × 20	79.3	III 60^b^ IV 7
Laramore [69]	13	40–50	EBRT	40–50	61.5	III 0 IV 0
Phromratanapongse [71]	44	35–66	EBRT + HT	16–56	40.9	III 25 IV NR
Li [80]	41	58	EBRT	43	56	IV 8
Jones^a^ [60]	52 56	NR	EBRT EBRT + HT	30–66 60–70	42.3 66.1	III 2 III 5
Kouloulias [81]	15	60	EBRT + HT	30.6	20	III NR IV 7
Linthorst [73]	198	48	EBRT + HT	8 × 4	78^c^	III/IV 11.9^d^
Linthorst [78]	248	49	EBRT + HT	8 × 4	39^c^	III 1^d^

*BT* brachytherapy, *HT* hyperthermia, *EBRT* external beam radiotherapy, *NR* not reported

^a^Breast cancer and other tumor entities

^b^Predominantly telangiectasia

^c^5-year local control rate

^d^5-year rate

#### Conclusions of the DEGRO expert panel for the therapy of locoregional recurrences after mastectomy

- Multimodality therapy including systemic therapy, surgery, and radiation +/− hyperthermia achieves a high rate of local control and can be curative with long-term survival in a subset of patients.
- Patients with an isolated locoregional recurrence after mastectomy should undergo surgical resection. Postoperative radiation therapy to the chest wall is mandatory and regional nodal irradiation (RNI) is strongly advised.
- In radiation-naive patients, the chest wall and regional lymph nodes should be treated with doses of 50–50.4 Gy (1.8–2 Gy per day). A boost dose of 10 Gy may be applied. Further dose escalation does not seem to improve treatment results.
- In previously irradiated patients with a high risk of a second local recurrence after surgical resection or in patients with unresectable recurrences, reirradiation should be strongly considered. Indication and dose concepts depend on the time interval to first radiotherapy, presence of late radiation effects, and concurrent or sequential systemic treatment.
- In the absence of severe radiogenic stigmata and an appropriate time interval (> 1 year), reirradiation with doses between 45 and 50 Gy is recommended, but should not exceed cumulative doses of 100–110 Gy_3_ (2-Gy_3_ equivalent dose).
- Particularly in previously irradiated patients, combination with hyperthermia can further improve tumor control.

## Regional recurrences and isolated supraclavicular lymph node recurrences

**Statement of the German S3 Guidelines 2012** [13]:

**Statement recurrence 3: Isolated regional recurrence**

- In case of an isolated regional recurrence, local control should be achieved with surgery/radiotherapy.

### Axillary recurrence

The reported cumulative risk of axillary recurrence after axillary dissection ranges from 0.5 to 3.0 % [82, 83]. Voogd et al. [82] investigated the long-term prognosis of 59 out of 4669 patients (1.3 %) with an axillary recurrence after axillary dissection. The median interval between treatment of the primary tumor and diagnosis of axillary recurrence was 2.6 years (range 0.3–10.7 years). Distant metastases occurred in 38 of the 59 patients. The 5- and 10-year distant recurrence-free survival rates were 39 % (95 % confidence interval, CI: 25–52 %) and 29 % (95 % CI: 16–42 %), respectively. The authors concluded that axillary recurrence following axillary dissection was associated with a high rate of subsequent distant metastasis and poor overall prognosis, although cure was still achievable in one third of the patients. Newman and colleagues [84] reported on 44 out of 4255 (1 %) breast cancer patients with axillary recurrence. With a median follow-up of 70.8 months, complete control of axillary recurrence was achieved in 31 patients (71 %). Distant metastases developed in 50 % and were more likely to occur in the case of uncontrolled axillary recurrences. Of the patients receiving multimodality therapy (75 %), the most common sequence was surgery followed by either systemic therapy and/or nodal irradiation. The addition of radiotherapy significantly improved axillary disease control (80.8 vs. 55.6 %).

### Isolated supraclavicular lymph node recurrence

Van der Sangen et al. [85] investigated 4669 patients treated for invasive breast cancer. An isolated supraclavicular lymph node recurrence developed in 42 patients (0.9 %). Different treatments, including surgery, radiotherapy, systemic therapy, or a combination of these, were applied. Complete remission was achieved in 35 patients (83 %). However, a second supraclavicular recurrence occurred in a third of these patients. Overall, 6 patients (14 %) were alive without evidence of disease after a follow-up period of 4.4–8.3 years. The 5-year actuarial OS and distant disease-free survival rates were 38 and 22 %, respectively. Distant disease-free survival was somewhat better in the 25 patients who underwent radiotherapy as part of the treatment for supraclavicular recurrence than it was in the 17 patients who did not (*p* = 0.06). The difference reached statistical significance after the exclusion of 8 patients who had received axillary and supraclavicular radiotherapy as part of their primary tumor treatment (*p* = 0.002). In summary, complete remission can be obtained in most patients with isolated supraclavicular recurrence, although the prognosis for these patients is poor.

#### Conclusions of the DEGRO expert panel for the treatment of axillary or supraclavicular lymph node recurrence

- Isolated axillary and supraclavicular recurrences from breast cancer are uncommon and may follow any stage of disease. Of the affected patients, 50–65 % develop distant metastases.
- To date, only retrospective data concerning the treatment of regional nodal recurrence are available.
- Durable disease control is best achieved with multimodality therapy including surgery and radiotherapy. Approximately one third of patients with an axillary breast cancer recurrence can be cured with multimodal therapy.
- Radiation therapy significantly improves local control and should be applied whenever feasible.
